# Community perspectives on the COVID-19 response, Zimbabwe

**DOI:** 10.2471/BLT.20.260224

**Published:** 2020-10-28

**Authors:** Constance RS Mackworth-Young, Rudo Chingono, Constancia Mavodza, Grace McHugh, Mandikudza Tembo, Chido Dziva Chikwari, Helen A Weiss, Simbarashe Rusakaniko, Sithembile Ruzario, Sarah Bernays, Rashida A Ferrand

**Affiliations:** a Department of Global Health and Development, Faculty of Public Health and Policy, London School of Hygiene and Tropical Medicine, 15–17 Tavistock Place, London, WC1H 9SH, England.; b Biomedical Research and Training Institute, Harare, Zimbabwe.; c Department of Public Health, Environments and Society, London School of Hygiene and Tropical Medicine, London, England.; d MRC Tropical Epidemiology Group, London School of Hygiene and Tropical Medicine, London, England.; e Department of Clinical Research, London School of Hygiene and Tropical Medicine, London, England.; f Community Medicine Department, University of Zimbabwe, Harare, Zimbabwe.; g Medical Research Council of Zimbabwe, Harare, Zimbabwe.; h School of Public Health, University of Sydney, Sydney, Australia.

## Abstract

**Objective:**

To investigate community and health-care workers’ perspectives on the coronavirus disease 2019 (COVID-19) pandemic and on early pandemic responses during the first 2 weeks of national lockdown in Zimbabwe.

**Methods:**

Rapid qualitative research was carried out between March and April 2020 via phone interviews with one representative from each of four community-based organizations and 16 health-care workers involved in a trial of community-based services for young people. In addition, information on COVID-19 was collected from social media platforms, news outlets and government announcements. Data were analysed thematically.

**Findings:**

Four themes emerged: (i) individuals were overloaded with information but lacked trusted sources, which resulted in widespread fear and unanswered questions; (ii) communities had limited ability to comply with prevention measures, such as social distancing, because access to long-term food supplies and water at home was limited and because income had to be earned daily; (iii) health-care workers perceived themselves to be vulnerable and undervalued because of a shortage of personal protective equipment and inadequate pay; and (iv) other health conditions were sidelined because resources were redirected, with potentially wide-reaching implications.

**Conclusion:**

It is important that prevention measures against COVID-19 are appropriate for the local context. In Zimbabwe, communities require support with basic needs and access to reliable information to enable them to follow prevention measures. In addition, health-care workers urgently need personal protective equipment and adequate salaries. Essential health-care services and medications for conditions other than COVID-19 must also continue to be provided to help reduce excess mortality and morbidity.

## Résumé

**Objectif:**

Connaître le point de vue des communautés et soignants vis-à-vis de la pandémie de maladie à coronavirus 2019 (COVID-19) ainsi que des réactions initiales durant les deux premières semaines de confinement au Zimbabwe.

**Méthodes:**

Une recherche qualitative rapide a été menée entre mars et avril 2020 sous forme d'entretiens téléphoniques avec un représentant de chacun des quatre organismes communautaires et 16 soignants qui participaient à un test sur les services communautaires destinés aux jeunes. En outre, des informations sur la COVID-19 ont été récoltées via les réseaux sociaux, les médias et les déclarations du gouvernement. Les données ont été analysées par thèmes.

**Résultats:**

Quatre thèmes ont été identifiés: (i) les individus étaient submergés d'informations mais manquaient de sources fiables, ce qui a engendré de la peur et laissé de nombreuses questions en suspens; (ii) les communautés disposaient de capacités limitées pour appliquer les mesures de prévention telles que la distanciation physique, en raison d'un accès restreint à l'approvisionnement alimentaire et à l'eau à domicile et de la nécessité de générer des revenus au quotidien; (iii) les soignants se considéraient comme vulnérables et peu reconnus, compte tenu de la pénurie d'équipements de protection personnelle et de l'insuffisance de leur rémunération; et enfin, (iv) d'autres pathologies ont été négligées car les ressources étaient réaffectées, avec des répercussions parfois considérables.

**Conclusion:**

Les mesures de prévention pour lutter contre la COVID-19 doivent impérativement être adaptées au contexte local. Au Zimbabwe, les communautés requièrent du soutien pour répondre aux besoins fondamentaux et accéder à des informations fiables qui leur permettent d'appliquer ces mesures de prévention. D'autre part, les soignants doivent de toute urgence disposer d'un équipement de protection personnelle et bénéficier d'une rémunération adéquate. Les médicaments et soins de santé essentiels pour des pathologies autres que la COVID-19 doivent également continuer à être administrés pour contribuer à diminuer les taux de mortalité et de morbidité excessifs.

## Resumen

**Objetivo:**

Examinar las opiniones de la comunidad y de los profesionales sanitarios sobre la pandemia de la enfermedad por coronavirus de 2019 (COVID-19) y sobre las respuestas tempranas a la pandemia durante las dos primeras semanas de confinamiento nacional en Zimbabue.

**Métodos:**

Entre marzo y abril de 2020 se realizó un estudio cualitativo rápido mediante entrevistas telefónicas con un representante de cada una de las cuatro organizaciones comunitarias y con 16 profesionales sanitarios que participaban en un estudio sobre los servicios comunitarios para jóvenes. Además, se recopiló información sobre la COVID-19 a través de plataformas de las redes sociales, los medios de comunicación y los anuncios del gobierno. Los datos se analizaron temáticamente.

**Resultados:**

Surgieron cuatro temas: i) las personas estaban sobrecargadas de información y no disponían de fuentes fiables, lo que generaba un temor generalizado y dudas sin respuestas; ii) las comunidades tenían una capacidad limitada para cumplir las medidas de prevención, como el distanciamiento físico, debido a que el acceso a los suministros de alimentos y de agua a largo plazo en el hogar era limitado, además de que debían percibir ingresos diariamente; iii) los profesionales sanitarios se consideraban vulnerables y subestimados debido a la escasez de los equipos de protección personal y a una remuneración inadecuada; y iv) se excluían otras enfermedades debido a la reorientación de los recursos, lo que planteaba riesgos importantes.

**Conclusión:**

Es importante que las medidas de prevención contra la COVID-19 sean apropiadas para el contexto local. En Zimbabue, las comunidades necesitan apoyo para satisfacer sus necesidades básicas y tener acceso a información fiable que les permita aplicar las medidas de prevención. Además, los profesionales sanitarios necesitan con urgencia equipos de protección personal y remuneraciones adecuadas. También se deben seguir prestando los servicios básicos de atención sanitaria y los medicamentos para las enfermedades distintas de la COVID-19 para contribuir a la reducción del exceso de mortalidad y morbilidad.

## ملخص

الغرض استقصاء وجهات نظر المجتمع والعاملين في مجال الرعاية الصحية حول جائحة مرض فيروس كورونا 2019 (كوفيد 19)، والاستجابات المبكرة للجائحة خلال الأسبوعين الأولين من الإغلاق الوطني في زمبابوي.

الطريقة تم إجراء بحث نوعي سريع بين مارس/آذار وأبريل/نيسان 2020 عبر مقابلات هاتفية مع ممثل واحد لكل منظمة من منظمات مجتمعية أربعة، كما شارك 16 عاملاً في مجال الرعاية الصحية في تجربة الخدمات المجتمعية للشباب. بالإضافة إلى ذلك، تم جمع معلومات حول كوفيد 19 من منصات وسائل التواصل الاجتماعي، ومصادر الأخبار والإعلانات الحكومية. وتم تحليل البيانات بشكل موضوعي.

**النتائج برزت أربعة موضوعات:**

(1) كان الأفراد مثقلون بالمعلومات ولكنهم كانوا يفتقرون إلى المصادر موثوقة، وهو ما أدى إلى انتشار الخوف والأسئلة دون إجابة؛ و(2) كان لدى المجتمعات قدرة محدودة على الالتزام بتدابير الوقاية، مثل التباعد الاجتماعي، وذلك لأن الوصول إلى الإمدادات الغذائية طويلة الأجل والمياه في المنزل كان محدودًا، ولأن الدخل كان يجب كسبه يوميًا؛ و(3) أدرك العاملون في مجال الرعاية الصحية أنهم معرضون للخطر، وغير مُقدّرين حق تقديرهم بسبب النقص في معدات الحماية الشخصية والأجور غير الكافية؛ و(4) تم تهميش الظروف الصحية الأخرى بسبب إعادة توجيه الموارد، مما قد يكون له آثار واسعة النطاق.

الاستنتاج من الهام أن تكون تدابير الوقاية من كوفيد 19 مناسبة للأوضاع المحلية. وفي زمبابوي، تتطلب المجتمعات إلى دعم يتعلق بالاحتياجات الأساسية والوصول إلى المعلومات الموثوقة لتمكينها من اتباع تدابير الوقاية. بالإضافة إلى ذلك، يحتاج العاملون في مجال الرعاية الصحية بشكل عاجل إلى معدات الحماية الشخصية ورواتب مناسبة. يجب أيضًا الاستمرار في تقديم خدمات الرعاية الصحية الأساسية والأدوية للحالات الأخرى غير كوفيد 19، للمساعدة في الحد من معدلات الوفيات والمراضة الزائدة. ****

## 摘要

**目的:**

调查社区和卫生保健工作者对 2019 冠状病毒病（新型冠状病毒肺炎）疫情爆发情况以及津巴布韦在前两周实施全国性封锁期间对早期疫情采取的应对措施的看法。

**方法:**

在 2020 年 3 月至 4 月之间开展快速定性研究，从四个社区组织中分别选择一名代表并选择参与针对年轻人的社区服务试验的 16 名卫生保健工作者进行电话采访。此外，还通过社交媒体平台、新闻媒体和政府公告收集了新型冠状病毒肺炎相关信息。按主题分析了数据。

**结果:**

共分为四个主题：(i) 个人信息过多，但缺乏可靠来源，从而引起大范围恐慌且积累了大量问题无人解答；(ii) 社区在落实预防措施的能力有限，例如保持社交距离，因为隔离在家难以保证粮食和水资源的长期供应，且每天必须赚取收入；(iii) 由于缺乏个人防护设备且薪资过低，卫生保健工作者认为自己易受病毒攻击且不被重视；以及 (iv) 因为资源被重新分配，其他健康问题被搁置，从而可能产生广泛影响。

**结论:**

保证应对新型冠状病毒肺炎的预防措施与当地情况相符，这点非常重要。在津巴布韦，社区须获得基本需求相关支持并获取可靠信息，以确保其能够遵守预防措施。此外，迫切需要解决卫生保健工作者的个人防护设备和适当薪水问题。还必须继续为新型冠状病毒肺炎以外的疾病提供基本的医疗保健服务和药物，以降低过高的死亡率和发病率。

## Резюме

**Цель:**

Изучить мнение местного населения и медицинских работников о пандемии коронавирусного заболевания 2019 года (COVID-19) и ранних мерах противодействия распространению пандемии в течение первых двух недель общенационального карантина в Зимбабве.

**Методы:**

Экспресс-исследование качественных показателей проводилось в период с марта по апрель 2020 года посредством телефонных опросов с участием одного представителя от каждой из четырех общинных организаций и 16 работников здравоохранения, участвовавших в пробном проекте оказания общественных услуг молодежи. Кроме того, информация о COVID-19 собиралась из социальных сетей, сообщений новостных агентств и заявлений правительства. Данные анализировались методом тематического анализа.

**Результаты:**

Были выявлены четыре темы: i) люди были перегружены информацией, но не имели надежных источников информации, что приводило к повсеместному распространению страха и многочисленным вопросам, ответы на которые получить было невозможно; ii) общины имели ограниченные возможности для реализации мер противодействия распространению вируса, таких как социальное дистанцирование, поскольку доступ к долгосрочным запасам продовольствия и воды в домах был ограниченным, а доход должен был обеспечиваться на ежедневной основе; iii) медицинские работники считали себя уязвимыми и недооцененными по причине нехватки средств индивидуальной защиты и неадекватной оплаты труда; iv) иные состояния и заболевания были отодвинуты на второй план по причине перенаправления ресурсов, что было чревато серьезными последствиями.

**Вывод:**

Важно обеспечить соответствие мер профилактики распространения COVID-19 локальным условиям. Общинам Зимбабве требуется поддержка в удовлетворении основных потребностей и доступ к достоверной информации, что позволит им обеспечить соблюдение профилактических мер. Кроме того, необходимо срочно обеспечить медицинских работников средствами индивидуальной защиты, а также адекватной заработной платой. Необходимо также обеспечить предоставление основных медицинских услуг и препаратов для лечения иных заболеваний, помимо COVID-19, чтобы способствовать снижению чрезмерной смертности и заболеваемости.

## Introduction

In early 2020, the coronavirus disease 2019 (COVID-19) pandemic began sweeping across the globe and threatened to profoundly affect sub-Saharan Africa.^1^ Many high- and middle-income countries imposed stern restrictions to facilitate social distancing and health system preparedness.^2^ Similar policies were swiftly implemented in low-income countries. However, these control measures presented acute challenges for many sub-Saharan African countries where a weakened infrastructure, overstretched health systems and limited public health surveillance compromised their potential efficacy.^3^ In Zimbabwe, periodic strikes by health-care workers in protest against under-resourcing of the health system and low salaries further limited the capacity to respond.

The pandemic was expected to have a wide-reaching, social, economic and health impact in low- and middle-income countries despite their generally younger populations.^4^^,^^5^ Three factors threatened morbidity and mortality rates in these countries: (i) overcrowding and large household sizes, which could increase transmissibility; (ii) the high prevalence of comorbidities, which could make progression to severe disease more likely; and (iii) the lack of intensive care capacity, which could increase case fatality rates.^4^ In addition, the Ebola virus disease outbreak 2013–2016 showed that, “the indirect mortality effects of a crisis in… a health system lacking resilience may be as important as the direct mortality effects...”.^6^

On 28 March 2020, the Zimbabwean government announced a national lockdown to be implemented within 48 hours. Initially, all nonessential businesses had to close and all citizens had to remain in their homes for 21 days.^7^ Only essential activities, defined as purchasing basic necessities, going to work (if employed by an essential service provider) and going to a relative’s house to provide care, were allowed outside the home.

Like many countries in sub-Saharan Africa, Zimbabwe has an under-resourced health-care system, high unemployment, densely populated urban areas and shortages of basic commodities (including water and food),^8^ which make lockdowns difficult to adhere to and enforce. By early 2020, little research had been carried out on how to adapt COVID-19 pandemic responses to local settings in sub-Saharan Africa. Lessons from responses to Ebola virus disease and human immunodeficiency virus (HIV) infections highlighted the pivotal influence of community engagement in decision-making and in devising locally affordable and effective prevention measures.^9^^–^^11^ Often, however, community engagement is only taken seriously after other epidemiological efforts have failed to stem infection rates.^12^^,^^13^

In our view, community engagement should be an integral pillar of efforts to address COVID-19 in sub-Saharan Africa from the start, rather than an afterthought. This pillar should include openness to feedback from the community and community leaders.^10^^,^^14^ We studied community and health-care workers’ perspectives on COVID-19 and on early pandemic responses in the first 2 weeks of the Zimbabwean lockdown, which necessarily situated our findings within this phase of the pandemic. Our findings, which were presented to the Zimbabwean Ministry of Health and Childcare and the COVID-19 Taskforce in April 2020, helped inform decisions on pandemic prevention strategies in the country.

## Methods

We investigated the social impact of the COVID-19 epidemic in Zimbabwe by rapidly conducting qualitative research that drew on existing studies and networks. We used three data sources: (i) phone interviews with representatives of four community-based organizations; (ii) phone interviews with 16 community health workers, nurses, sexual and reproductive health counsellors, and youth workers; and (iii) the collation of rumours and information about COVID-19 from social media, news outlets and government announcements. Data were collected remotely to avoid physical contact.

### Community-based organizations

Community-based organizations in Chitungwiza, a city near the capital Harare, were asked to participate through convenience sampling. One representative from each of four organizations was interviewed by phone on how they and their organizations were affected by, and responding to, the COVID-19 epidemic. Three representatives were women (age range: 25 to 50 years). Interview topics included: (i) personal perceptions of COVID-19 in Zimbabwe; (ii) their organization’s response; (iii) community sources of influential information; (iv) community perceptions and behaviours; and (v) personal perceptions of social isolation policies. Verbal consent to recording the interviews was obtained. The method of summarizing recorded interviews has been described previously.^15^

### Health-care workers

We conducted phone interviews with seven community health workers, five nurses, two sexual and reproductive health counsellors, and two youth workers involved in the CHIEDZA trial,^16^ which is a cluster randomized trial of community-based, sexual and reproductive health and HIV services for young people in three provinces: Bulawayo, Harare and Mashonaland East. Interviews were conducted during the first 2 weeks of the government-mandated lockdown, when the trial had been suspended. Ten of the 16 health-care workers were women (age range: 24 to 55 years). Interview topics included: (i) changes in health service delivery; (ii) health-care workers’ concerns; (iii) the impact of COVID-19 on the provision of other health services; and (iv) the impact of COVID-19 on their personal lives. Phone interviews were recorded and transcribed.

### Rumours and information about COVID-19

Six researchers collated COVID-19 information (including rumours and myths) provided via social media platforms (i.e. WhatsApp, Twitter, Facebook and Instagram), local and international news outlets, and government and nongovernmental organization announcements. We developed a table to guide information gathering, summarizing and synthesis. Information from 147 WhatsApp messages, social media videos, government announcements and other sources were collated in a single document.

### Data analysis

The lead data collector for each data source presented a summary of the key analytical findings to the research team and four themes commonly observed across all three data sources were identified in discussions. Data were manually and thematically coded according to these four themes, then data relating to each theme were extracted and compared across data sources. In this process, subthemes were identified inductively. Ethical approval was obtained from the Medical Research Council of Zimbabwe (MRCZ/A/2387).

## Results

We identified four thematic areas: (i) information overload but a lack of trusted sources; (ii) communities’ limited ability to comply with prevention measures; (iii) health-care workers’ perceived personal vulnerability; and (iv) sidelining of other health issues.

### Information overload but few trusted sources

Participants reported being bombarded with information about COVID-19 from, for example: (i) social media (mostly WhatsApp but also Facebook, Twitter and YouTube); (ii) radio; (iii) the Zimbabwe Broadcasting Cooperation; (iv) government announcements; (v) relatives living abroad; and (vi) conversations with neighbours (e.g. when queuing for water at boreholes). Nevertheless, there were many unanswered questions:

“I still feel like people have so many questions, they want answers in layman’s language. Like how it’s spread? What is it exactly? How can we stop it?” (female community health worker aged 26 years).

Although information was perceived as important for understanding and practising preventive measures, participants talked about “hearing many different myths” (man aged 49 years, community-based organization). Many were unsure about which sources to trust: “People are forwarding dangerous and toxic information which might not be true sometimes” (female community health worker aged 32 years). Even some government information was considered unreliable; there was a widespread view it was likely to have been censored and aimed at maintaining government interests.

Some rumours fuelled fear among communities and health-care workers. For example, there was a perception that COVID-19 was “more lethal than any other disease: cholera, Ebola” (woman aged 29 years, community-based organization). Conversely, other rumours encouraged a perception of immunity: “the virus cannot affect black people.” There was also the view that certain precautions, such as consuming bleach or lemon with sodium bicarbonate, could prevent infection. Perceptions of immunity or reduced risk may have encouraged noncompliance with prevention measures in communities.

### Communities’ limited ability to comply

In the first week after the government announcement that all nonessential businesses should close and no-one should leave their house, most individuals in communities like Chitungwiza continued their lives and social interactions as normal and there was no noticeable difference in the volume of people movements. Limited access to water and shortages of mealie meal (the staple food) – ongoing challenges in Zimbabwe – limited individuals’ capacity to stay indoors and practise social distancing:

“The one-meter apart rule, the funny thing, when I bought my mealie-meal at [a shop] in town, it was being practised inside the shop. But outside the shop, we were queued chest-to-chest, like bumper-to-bumper, you know” (female counsellor, aged 41 years)

People generally stayed within their localities but groups were “going to queue at the boreholes” (woman aged 48 years, community-based organization). Without pumped water to households or long-term food supplies, there was a tension between public health advice and the difficulty communities had in following that advice:

“Social distancing and hygiene are preached, but there is no way they can be practised when people are lacking such basic commodities” (woman aged 29 years, community-based organization).

Moreover, as income tended to be generated on a daily basis, “people survive hand to mouth” (woman aged 48 years, community-based organization) and had to purchase their food each day. Staying at home was impossible because it threatened peoples’ ability to meet their basic needs:

“they are thinking: what am I going to be feeding my children?” (woman aged 29 years, community-based organization). In addition, staying at home may also have increased other vulnerabilities, such as gender-based violence when individuals were “stuck in houses with people who are abusing them” (woman aged 29 years, community-based organization).

People were also frustrated that the government was not providing support to enable them to comply with prevention measures: “The government said there would be availability of power, or water, but in the community, nothing has improved. There is nothing. People have to go out of their homes in search of water. What they are saying is not what is happening on the ground” (man aged 49 years, community-based organization). 

Although government restrictions were the same for everyone, the ability to abide by them was clearly economically determined. Individuals thought there was a hierarchy within communities shaped by financial resources that influenced who was able to follow prevention measures and protect themselves. Being able to stay indoors was described as a privilege only wealthier individuals and communities could afford:

“People in my neighbourhood are a bit wealthier and can afford to buy more food. …we don’t need to go to boreholes, we’ve got wells and tanks. A lot of people here are upholding the social distancing thing” (woman aged 29 years, community-based organization).

### Health-care worker vulnerability

Health-care workers were fearful about their own infection risk and about the risk of infecting others. This fear existed despite workers’ understanding that the risk they would become infected or die was still relatively low: “You try to comfort yourself that you are not yet old, you are young, and COVID is not yet in Bulawayo. You know all those things” (female community health worker aged 32 years). However, this reasoning was undermined by the heightened risk of occupational exposure as workers were expected to do their jobs with a shortage of personal protective equipment:

“This is something that instils fear in us because at the end of the day you have to work, you have no option, but we don’t have the essential protective clothing required of us to use” (male community health worker aged 26 years).

Since September 2019, health-care workers in Zimbabwe, including doctors and nurses, have been engaged in intermittent strike action to demand greater resources for the health system and increases in low salaries and allowances. On 25 March 2020, the Zimbabwe Nurses Association went on strike because of a lack of personal protective equipment, unreliable water supplies and the need for a COVID-19 risk allowance. Workers felt undervalued and exposed, which culminated in a lack of trust that the health-care system could protect them from COVID-19: “I was talking about Harare Central Hospital, and it’s not even functioning and nurses have just downed their tools and the doctors as well” (female nurse aged 44 years). The lack of available nurses was already having negative consequences at the time of data collection:

“We had Ruth’s (pseudonym) father who passed away. He had had instruction to get an operation, but I think it couldn’t happen with the go-slow because people had downed their tools... It took death to a patient to reveal that he didn’t matter” (female nurse aged 44 years).

### Sidelining of other health issues

Participants were concerned that a narrow focus on preventing and treating COVID-19 would lead to the needs of people having other diseases being neglected. Several participants noted this was already happening as patients were being “turned away at pharmacies because of this coronavirus” (woman aged 48 years, community-based organization). It was predicted that the incidence of sexually transmitted diseases and HIV infection would increase without prevention measures, such as condoms. In particular, an increase in unintended pregnancies due to impeded access to family planning was predicted:

“Look at family planning: there are some who are due to have their depo injections resupplied every 3 months. And they are due and they can’t even get the depo because the city council clinics, I don’t think they have it. And they don’t have the money to buy themselves” (female nurse aged 44 years).

In response to the COVID-19 pandemic, views of what comprised essential medicines and medical procedures were reframed and many procedures were deprioritized. This response had implications for people with other diseases and increased the vulnerability to COVID-19 of those with comorbidities. Moreover, a lack of hospital transport and roadblocks created additional difficulties for people with chronic diseases and for those with COVID-19 symptoms who needed urgent care:

“We have got people who are on dialysis and they need to be taken to the clinic or hospitals for their dialysis and they will not be able to do that because there is no public transportation. That is also going to make them deteriorate.” (female community health worker aged 36 years).

However, attempts have been made to mitigate the effect of limited access to medications. For example, participants described efforts to provide 3 or 6 months’ supply of antiretroviral treatment in advance to people living with HIV. Thus, the health-care system exhibited some agility in responding pre-emptively to challenges. Nevertheless, despite these efforts, people with HIV infections were prevented from accessing treatment at clinics by a lack of transport and roadblocks, which led to “forced nonadherence” (female community health worker aged 32 years).

## Discussion

Our study reports the social, financial and resource-related obstacles encountered in implementing COVID-19 prevention measures in Zimbabwe when the country first went into national lockdown in March and April 2020, which had a devastating effect on livelihoods.^17^ The collateral damage of COVID-19 in the country is extensive as the weakened health system struggles to manage the intertwined threats of health-care worker strikes and COVID-19.^18^^,^^19^

Our findings confirm fears that social distancing and hand hygiene measures may not be feasible in sub-Saharan African countries, particularly in lower-income communities where water is often available only at public boreholes and income is earned informally on a day-to-day basis, thereby necessitating daily food purchasing.^3^^,^^20^ Compliance with social distancing and lockdown measures in Zimbabwe could be improved by providing support packages for families and communities. Reinstating water supplies to homes, distributing food packages and providing cash transfers could both offset the economic damage from COVID-19 and enable families to stay at home. Support could be facilitated by government partnerships with international organizations.^21^

The development of efficacious preventive measures that were acceptable to local communities was critical in the early stage of the pandemic and remains so. According to one commentator, there is a need to think “about solutions that are not based on the legitimate fears of other nations, but on our own established realities.”^20^ Some locally relevant interventions were proposed. For example, the World Health Organization’s guidance on home-based care for patients with mild symptoms remains valuable, especially where health systems are overburdened and hospital-based isolation is not feasible.^22^ In addition, researchers have suggested that household- or community-based shielding is an option for high-risk individuals where social distancing is not possible: a room within a household or an area within a community could be allocated for these individuals.^4^ Mask-wearing, accompanied by messaging about their use with other prevention measures, has been adopted in many settings, particularly indoor communal settings.^23^^–^^25^

Our study highlighted the urgent need for sufficient personal protective equipment and adequate hand hygiene facilities in Zimbabwe to protect health-care workers from the elevated risk of infection and death.^26^ The World Health Organization recommends four items for those in contact with patients: gloves, face masks, gowns or aprons, and eye protection.^27^ Additionally, with protection, health-care workers feel more valued and have greater trust in the national response, as demonstrated in recent Ebola virus disease outbreaks.^28^ International donors could help supply personal protective equipment for health-care workers in low- and middle-income countries.^29^ Continued access to key health-care services, such as essential medicines, family planning and antiretroviral drugs, was also found to be important. The provision of additional resources early in the pandemic could have helped protect essential health-care services and reduce excess mortality and morbidity from other diseases,^3^ as observed in Sierra Leone during the Ebola virus disease epidemic, where excess maternal and newborn mortality equalled mortality due to the Ebola virus disease outbreak itself.^6^

There is a need for public health messages on behavioural change to be adapted to suit different community groups.^30^^,^^31^ For example, in Zimbabwe, advice could be provided on hygiene measures that can be taken when pumped water is not available in households. Such advice was noticeably lacking at the beginning of the pandemic and substantial gaps still remain. Public confidence in government messaging could have been improved by coordinating messages through an information centre similar to the coronavirus resource portal in South Africa.^32^ Support from publicly trusted, United Nations agencies would also boost confidence. Messaging needs to strike a delicate balance: it must communicate information about risk and the importance of complying with prevention measures without propagating fear, particularly where individuals will incur personal losses by complying with social isolation measures. Lessons from HIV responses show it is vital to engage communities, including community and faith leaders, from the beginning to build trust, to ensure interventions are effective and to enable the information provided to be reliable and reflect changes in knowledge of, and responses to, the epidemic.^10^

Our study’s findings are based on the experiences of people working in community-based organizations in Zimbabwe and of health-care workers employed on an existing trial. We acknowledge there may be limitations in extending our findings to workers in public facilities. Further qualitative research is being conducted with a broader range of community stakeholders and government-employed, health-care workers in Zimbabwe to provide more detailed understanding of community perceptions and the social impact of COVID-19.

Our study confirms the importance of taking community perspectives into account when designing locally effective interventions during a pandemic. Recommendations for COVID-19 public health measures in Zimbabwe are summarized in Box 1. These recommendations may also be relevant to other countries in the region, with adaptations to local conditions. Moreover, they could help strengthen health systems over the long term.

Box 1 **Recommended public health measures for the COVID-19 pandemic in Zimbabwe**

- Provide support for households to enable families to stay at home, such as reinstating household water supplies, distributing food packages and making cash transfers.
- Support research into, and the development of, locally appropriate prevention measures for COVID-19 (and other infectious diseases), such as improvements in water supplies and hygiene facilities.
- Provide sufficient personal protective equipment and hand hygiene facilities for health-care workers.
- Continue to provide health-care services, essential medicines and preventive methods for conditions other than COVID-19 and ensure health-care workers receive adequate remuneration.
- Disseminate coherent, accurate, trusted and targeted information to the public and all stakeholders.

COVID-19: coronavirus disease 2019.
